# Apoptosis in Pneumovirus Infection

**DOI:** 10.3390/v5010406

**Published:** 2013-01-22

**Authors:** Elske van den Berg, Job B.M. van Woensel, Reinout A. Bem

**Affiliations:** Pediatric Intensive Care Unit, Emma Children’s Hospital, Academic Medical Center, Meibergdreef 9, 1105 AZ Amsterdam, The Netherlands; E-Mails: elske.vandenberg@amc.nl (E.vd.B); j.b.vanwoensel@amc.nl (J.B.M.vW); r.a.bem@amc.nl (R.A.B)

**Keywords:** respiratory syncytial virus, cell death, host defense, acute lung injury

## Abstract

Pneumovirus infections cause a wide spectrum of respiratory disease in humans and animals. The airway epithelium is the major site of pneumovirus replication. Apoptosis or regulated cell death, may contribute to the host anti-viral response by limiting viral replication. However, apoptosis of lung epithelial cells may also exacerbate lung injury, depending on the extent, the timing and specific location in the lungs. Differential apoptotic responses of epithelial cells *versus* innate immune cells (e.g., neutrophils, macrophages) during pneumovirus infection can further contribute to the complex and delicate balance between host defense and disease pathogenesis. The purpose of this manuscript is to give an overview of the role of apoptosis in pneumovirus infection. We will examine clinical and experimental data concerning the various pro-apoptotic stimuli and the roles of apoptotic epithelial and innate immune cells during pneumovirus disease. Finally, we will discuss potential therapeutic interventions targeting apoptosis in the lungs.

## 1. Introduction

Pneumoviruses are single-stranded, negative-sense, enveloped RNA viruses belonging to the family *Paramyxoviridae*, subfamily *Pneumovirinae*, and include several closely related, but species-limited, members (reviewed by Easton *et al*. [1]). The human pneumovirus respiratory syncytial virus (hRSV) is a leading respiratory pathogen in young children and the elderly worldwide and is associated with considerable morbidity and mortality and high health care costs [2,3]. Likewise, bovine RSV (bRSV) causes outbreaks of respiratory disease in young beef and dairy cattle. Both bRSV infection in cattle and infection of mice by the rodent-specific pneumovirus pneumonia virus of mice (PVM) have been studied extensively as a model for hRSV disease in humans [4]. Pneumovirus infections in humans and animals cause a wide spectrum of respiratory disease symptoms, ranging from mild upper airway illness, such as coryza and cough, to lower respiratory tract disease (e.g., bronchiolitis and bronchopneumonia), which may eventually lead to impaired gas-exchange and life threatening respiratory failure. Human infants with hRSV infection are prone to develop acute respiratory distress syndrome (ARDS), an acute-onset life threatening inflammatory lung condition associated with widespread lung injury [5,6]. Currently, as no specific treatment options for severe hRSV disease in humans exist, there is an ongoing research effort focusing on pneumovirus biology and host-interaction, ultimately to find novel therapies.

Apoptosis, a highly regulated energy-dependent type of cell death with distinct morphological and biochemical characteristics [7], is a basic biological response of cells to virus entry and replication [8]. While apoptosis of virus-infected cells may be an important first line host defense mechanism to limit pathogen replication and spread, many viruses have evolved strategies to evade and modulate intracellular pro-apoptotic signaling in the early replication phase [9,10]. Conversely, it has become clear that viruses may also *exploit* the cellular pro-apoptotic machinery in the formation and spread of infectious progeny virions in the late phase or in the elimination of immune cells, thereby evading host defense [10,11]. At the same time, from the host’s perspective, extensive pro-apoptotic signaling may be beneficial in attacking a virus, but may become devastating upon the occurrence of an overshoot in apoptosis, leading to widespread loss of infected and/or uninfected bystander structural cells. Such an unbalanced extensive apoptotic response is implicated in the pathogenesis of a wide variety of diseases, including the development of diffuse lung epithelial injury in ARDS [12]. Taken together, the outcome of apoptosis during viral infection for the host may depend on its extent, timing and cell-specificity.

The occurrence and potential role of apoptosis in pneumovirus infections have been investigated in both *in vivo* (human and animal) and *in vitro* studies. The main goal of this manuscript is to provide an overview of the existing literature on pro- and anti-apoptotic signaling in pneumovirus infections with a focus on lung (airway and alveolar) epithelial cells, neutrophils and macrophages as first line cellular responders to acute pneumovirus infection. Furthermore, we will speculate on future apoptosis-based pharmacological therapies in hRSV disease.

## 2. Apoptotic Signaling Pathways

Cell death in multicellular organisms occurs either by necrosis or apoptosis, however their strict distinction is somewhat artificial, because overlap of their characteristics and cellular pathways may occur [13,14,15]. Apoptosis is associated with membrane blebbing, cell breakdown into apoptotic bodies and fragmentation of DNA. “Classical” apoptosis refers to the activation of the caspase cascade, a family of intracellular substrate specific proteases of which the final executioner, caspase-3, is a commonly used marker for apoptosis. Non-classical apoptosis occurs independent of caspase activation and involves release of the flavoprotein apoptosis-inducing factor (AIF) from mitochondria. Galluzzi *el al*. have provided an extensive and detailed review on the use and interpretation of caspase-(in)dependent apoptosis assays in laboratory research [16].

**Figure 1:**
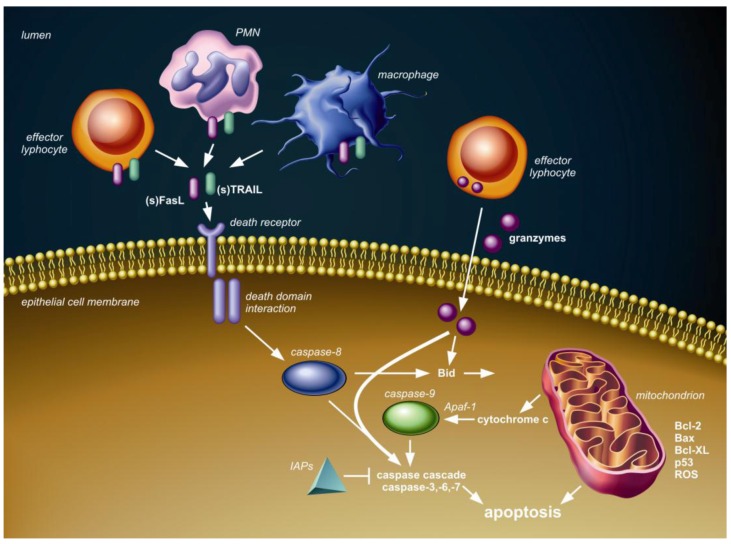
Schematic overview of three pathways of caspase-dependent apoptosis. First, the death receptor (extrinsic) pathway is activated upon tumor necrosis factor (TNF) death receptor family ligation by membrane-bound or soluble ligands, such as Fas ligand (FasL) and TNF-related apoptosis-inducing ligand (TRAIL), presented or secreted by local immune cells, including effector lymphocytes, neutrophils (PMN) and/or macrophages. Intracellular adaptor protein interactions through death domain modules follow the death receptor ligation and subsequently lead to activation of initiator caspase-8 and the downstream caspase cascade resulting in apoptosis. The inhibitor of apoptosis proteins (IAPs) can block several caspases, thereby inhibiting cell death. Second, granzymes delivered into the cytosol by effector lymphocytes can interact with several caspases and Bid to induce apoptosis. Third, members of the Bcl-2 family, including Bcl-2, Bax and Bcl-XL and p53, regulate cytochrome c release from the mitochondria (intrinsic pathway) in response to stimuli, such as DNA damage, infection and formation of reactive oxygen species (ROS). Cytochrome c in the cytosol assembles with apoptotic peptidase activating factor 1 (Apaf 1) to activate initiator caspase-9 with subsequent activation of the caspase-cascade and apoptosis. The mitochondrial and death receptor pathway can interact through BH3-interacting domain death agonist (Bid).

Figure 1 shows the conceptual framework of the major (caspase-dependent) apoptotic pathways, which are relevant to discuss in the context of pneumovirus infection. The intrinsic or mitochondrial pathway involves the release of cytochrome c from mitochondria into the cytoplasm and is regulated by Bcl-2 protein family members. The extrinsic or death receptor pathway is triggered by ligation of transmembrane death receptors that belong to the tumor necrosis factor (TNF) death receptor family, which include TNF receptor (TNFR), Fas (CD95) and TNF-related apoptosis-inducing ligand (TRAIL) death receptors (TRAIL-R1 and -R2). Expression and activation of proteins in both these pathways can be modulated by p53, a major cell stress sensor protein. Finally, the granule-mediated cytotoxic pathway is exploited by effector lymphocytes, which can release serine proteases, known as granzymes, into the cytosol of target cells, which subsequently results in caspase-(in)dependent apoptosis. Activation and modulation of these three apoptotic pathways is associated with pneumovirus infection in humans and animals, as will be discussed below.

## 3. Lung (Airway and Alveolar) Epithelial Cell Apoptosis

**Figure 2:**
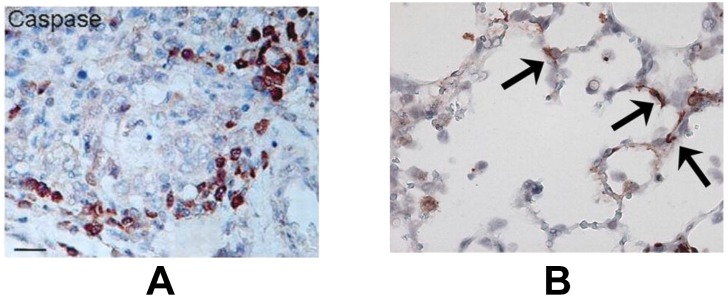
(**A**) Positive immunohistochemical staining for the apoptosis marker caspase-3 (brown) in bronchiolar epithelial cells in lung tissue from a child with fatal human pneumovirus respiratory syncytial virus (hRSV) disease. From Welliver *et al.* [24], by permission of Oxford University Press. (**B**) Positive immunohistochemical staining for caspase-3 (brown, arrows) in alveolar epithelial cells in lung tissue from a mouse (C57Bl/6 background) with severe pneumonia virus of mice (PVM) disease. From Bem *et al.* [25], Copyright 2010, The American Association of Immunologists, Inc.

In the lower airways, the bronchiolar epithelium is the primary site of human and animal pneumovirus infection [17,18,19,20]. In addition, viral antigen can be detected in alveolar epithelial cells in severe hRSV-, bRSV- and PVM-induced bronchopneumonia [18,19,20,21]. An interesting and consistently reported feature of severe pneumovirus disease is the sloughing of dead airway epithelial cells, which form dense plugs with mucus, fibrin and leukocytes, resulting in air trapping and ventilatory failure [18,20,21,22,23]. The loss of these epithelial cells has always been thought to occur primarily through necrosis, however Welliver *et al*. observed marked active caspase-3 immunostaining in bronchiolar epithelium of children with fatal hRSV disease, suggesting that apoptosis is an important mechanism of cell death as well (Figure 2A) [20,24]. Likewise, lung epithelial cells show enhanced DNA fragmentation as detected by terminal dUTP nick-end labeling (TUNEL) in bRSV infected calves and increased caspase-3 activation in PVM infected mice [19,25] (Figure 2B). The relative importance of apoptosis in cell decay in pneumovirus infected lungs is further supported by the finding of a strong correlation between caspase-3 and -7 activity and LDH concentration in the airways in children with hRSV-induced bronchiolitis; however, from this study, the exact cellular source of these markers is not clear [26].

**Figure 3:**
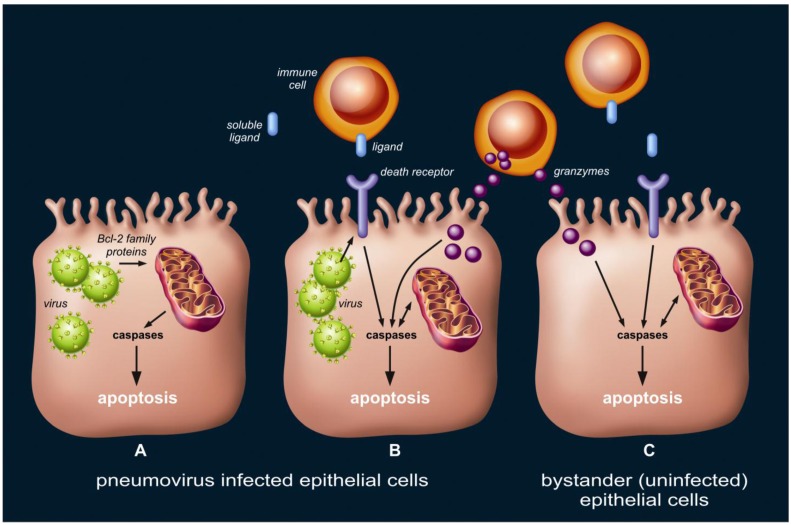
Three theoretical scenarios regarding lung epithelial cell apoptosis during pneumovirus infection, which may co-exist. First (**A**), viral infection triggers the mitochondrial (intrinsic) apoptotic pathway via interaction with Bcl-2 family proteins. Second (**B**), death receptor ligands (either membrane-bound or soluble) presented or secreted by local immune cells activate the death receptor (extrinsic) apoptotic pathway in viral-infected cells. Similarly, granzymes released from effector lymphocytes into the cytosol of target cells induce apoptosis. Viral infection may modulate the susceptibility to death receptor ligands or granzymes by altering the expression of and interaction with the protein machinery, such as surface death receptors, involved in these pathways. Third (**C**), bystander (uninfected) epithelial cells undergo apoptosis as a result of extensive, non-specific signaling via the death receptor (extrinsic) and/or granzyme apoptotic pathway.

The occurrence of apoptosis in the lung epithelium during the course of pneumovirus infection may serve to limit viral replication. However, the widespread and extensive scale on which this takes place, involving the whole pulmonary system, including the alveoli, may also suggests an overshoot and/or inefficiency of pro-apoptotic signaling during the late and severe phase of pneumovirus infection. This may lead to enhanced respiratory disease, e.g., diffuse alveolar epithelial injury, as seen in human ARDS [12] and influenza virus animal models [27]. We believe three scenarios regarding lung epithelial cell apoptosis during pneumovirus infection may co-exist as described below (Figure 3), and the balance between them is likely to be critical for the development and outcome of host disease.

### 3.1. Pneumovirus Infected Cells May Undergo Apoptosis as a Result of Direct Activation of Intrinsic Pathways.

Based on the results of a number of *in vitro *studies in cultured primary airway epithelial cells and A549 cells (an adenocarcinomic human alveolar basal epithelial cell line) it appears that, although in the early phase of hRSV infection apoptosis does not occur, this type of cell death becomes an important response in the later phase of viral replication [28,29,30,31,32,33,34,35,36,37,38,39]. It should be noted however that several of these studies show some conflicting results with regard to the time point at which apoptosis is detected, possibly related to the use of different cell lines/cultures, multiplicity of infection, hRSV strains, early versus late apoptosis markers and assays used.

The relative paucity of apoptosis during the early phase of hRSV infection may be explained by viral-induced anti-apoptotic intrinsic pathway signaling, which ensures more time for viral replication and assembly of virions. Mechanisms of early hRSV-induced alteration of the intrinsic pathway towards an anti-apoptotic balance in Bcl-2 family proteins include post-transcriptional regulation of p53 [32], microRNA modulation [40] and three important basic cell survival/proliferation pathways involving nerve growth factor/tyrosine kinase receptor (NGF/Trk) [36], epidermal growth factor receptor (EGFR) [35] and phosphoinositide 3-kinase(PI3K)/Akt signaling [38]. Interestingly, by using recombinant and RNA silencing techniques, it was shown that both the hRSV-encoded small hydrophobic (SH) protein and nonstructural (NS) proteins are critical regulators of the early anti-apoptotic effects [29,31]. The NS proteins have been found to interfere with interferon (IFN) signaling [41], which may affect apoptotic pathways in hRSV infected cells. However, Bitko *et al*. [29] suggested that the early NS protein associated anti-apoptotic effect was mediated through NF-κB and PI3K/Akt signaling, independent of the IFN pathway.

Pro-apoptotic signaling appears to predominate at a later phase of hRSV infection [29,30,34,39]. The hRSV-encoded fusion (F) protein, which is expressed at a later time point as compared to the NS proteins [29], has been found to be critical in the activation of apoptosis by p53-mediated activation of pro-apoptotic Bcl-2 family proteins [30]. Apoptosis markers in hRSV-infected A549 and human primary airway epithelial cells further include caspase activation, detection of phosphatidylserine exposition by Annexin V and TUNEL staining. However, the susceptibility of epithelial cells to pneumovirus-associated apoptosis may vary depending on the specific localization (proximal *versus* distal) in the lungs [34]. Importantly, in the recent study by Villenave *et al.* well-differentiated primary bronchial epithelial cells of children cultured in air-liquid interface showed TUNEL-positive staining in detached cells six days after hRSV infection [39]. This supports the hypothesis that apoptosis contributes to the characteristic sloughing of epithelial cells in the airways of children during the late phase of pneumovirus infection. 

### 3.2. Pneumovirus Infected Cells May Undergo Apoptosis by Activation of Extrinsic and Granule-Mediated Pathways

First, death receptor ligands may trigger apoptosis of virus infected epithelial cells. Several studies have shown enhanced local production of TNFα, FasL and TRAIL by recruited immune cells or neighbor epithelial cells upon pneumovirus infection [4,28,34,39,42,43]. Interestingly, *in vitro* studies have shown that, whereas hRSV infection results in decreased susceptibility to TNFα-induced apoptosis [44], it sensitizes lung epithelial cells to the pro-apoptotic effects of TRAIL [34], which likely occurs as a result of increased expression of death receptors TRAIL-R1 and -R2 [34,42]. Similarly, enhanced epithelial surface expression of Fas in hRSV infection has been found in both *in vitro* and human histopathology studies [20,28,45]. Interestingly, mice with a non-functional FasL (*gld* strain) have been reported to have delayed hRSV clearance [46], but van den Berg *et al. * [47] did not detect differences in lung viral loads in PVM-infected mice with a defective Fas (*lpr* strain) as compared to wild type mice. This differential effect of a dysfunctional Fas/FasL system on viral clearance in these different pneumovirus models may be explained by specific pneumovirus-host interactions, although may potentially also be related to a leaky, incomplete Fas defect in *lpr* mice.

Second, granzymes released from the granules of natural killer cells and cytotoxic T-lymphocytes into virus infected target cells may induce apoptosis. In children with severe hRSV disease, we have found strong release of granzymes in the lungs [48]. Similarly, PVM infected mice show enhanced granzyme expression, however this appears not to affect local viral titers [25], suggesting that the granule-mediated apoptotic pathway does not have an important role in clearance of pneumovirus infected epithelial cells.

### 3.3. Uninfected Bystander Epithelial Cells May Undergo Apoptosis as a Result of Extensive and Non-Specific Activation of Extrinsic and Granule-Mediated Pathways

Although pneumovirus antigen can be detected in alveolar epithelium in the late and severe phase of human and animal pneumovirus disease, the primary site of replication are (small) airway epithelial cells [17,18,19,20,21]. Interestingly, histopathology studies in humans with fatal hRSV disease and animal models show epithelial cell apoptosis may occur in the entire pulmonary system, including the alveolar compartment [4,19,24,25]. In fact, we have previously detected active caspase-3 immunostaining primarily in alveolar epithelial cells in mice with severe PVM disease, while this apoptosis marker was scarce in bronchial epithelium [25]. Such a potential disparity in the localization between pneumovirus presence and apoptosis may suggest uninfected bystander cells in the lungs are victim of an overshoot and/or inefficiency in pro-apoptotic signaling, and this may lead to enhanced pneumovirus disease.

The bystander injury hypothesis is supported by two *in vivo *studies in mice: Rutigliano *et al*. [46] showed that while a dysfunctional FasL results in delayed hRSV clearance, at the same time it significantly diminishes clinical illness; and, our own group showed that while a deficiency in granzymes did not affect PVM clearance, it resulted in delayed clinical disease in association with a marked decrease in lung caspase-3 activity [25]. In addition, we have previously shown that soluble TRAIL is present in increased levels in bronchoalveolar lavage fluid of children with severe hRSV disease, and this death receptor ligand induced cell death in uninfected primary pediatric airway epithelial cells *in vitro * [42]. Interestingly, similar findings for soluble FasL were found in relation to epithelial injury in human ARDS [49]. When considering the bystander epithelial injury hypothesis, soluble death receptor ligands present in the lung microenvironment during pneumovirus disease are of particular interest due to the potential increased likelihood of non-specific signaling. They may be released into the extracellular space from recruited immune cells, such as macrophages [42] and neutrophils [50] or from (infected) neighbor epithelial cells (paracrine pathway) [51], although *in vitro *studies by Bitko *et al*. [28] suggested a minor role for the latter mechanism.

## 4. Neutrophil Apoptosis

Neutrophils, recruited by local production of chemoattractants, are the predominant inflammatory cells in the pulmonary compartment during acute pneumovirus infection [4,52,53,54]. They possess a wide arsenal of defensive strategies against invading microorganisms, including phagocytosis, production of toxic reactive oxygen species and release of immune defense proteins. Although the role of neutrophils in anti-*viral* immunity remains relatively unclarified, several studies have shown a protective effect of neutrophils against influenza virus [55,56]. Both animal and human studies demonstrated that during pneumovirus infection, recruited neutrophils contain virus, suggesting a contributive role to viral clearance [19,57]. This is further supported by *in vitro* studies that showed neutrophil-mediated injury and detachment of hRSV infected lung epithelial cells [58]. On the other hand, it is well recognized that uncontrolled or prolonged neutrophil activity may cause collateral tissue injury [59], such as diffuse alveolar damage in human ARDS [60,61,62]. Although direct evidence of neutrophil-mediated respiratory disease in pneumovirus infection is lacking, the acute and strong neutrophil influx in the lungs coincides with peak disease severity in animal models [19,25].

To prevent their potential inadvertent harmful effect, the life span of neutrophils is limited by apoptosis. Interestingly, incubation of human neutrophils with hRSV *in vitro* results in activation of anti-apoptotic signaling through the Bcl-2 family proteins [63]. Although this inhibition of neutrophil cell death by hRSV does not require live virus, it does require cellular uptake. Lindemans *et al*. [63] further suggested this effect depends on auto- or paracrine-signaling via the release of IL-6. Others, however, have suggested that the anti-apoptotic effect of hRSV on human neutrophils is primarily mediated by soluble factors via monocyte interaction and, thus, is a secondary response [64]. Regardless of the precise mechanism involved, these *in vitro *studies suggest prolonged neutrophil survival in the setting of hRSV infection and, as such, this may be an important event in the development of lung injury during pneumovirus disease. In contrast, Wang *et al*. [65] reported an increased number of Annexin V positive neutrophils in nasopharyngeal aspirates and the peripheral blood of infants with hRSV disease, as compared to peripheral blood neutrophils of healthy children. This would imply that despite the pro-survival effect of hRSV on neutrophils as observed *in vitro*, pro-apoptotic signaling by mediators (e.g., cytokines) in the lung microenvironment during hRSV disease prevails. Whether this truly would protect against harmful prolonged neutrophil survival remains speculative. In particular, it is possible that different neutrophil apoptotic responses in functionally heterogeneous neutrophil subsets co-exist and change over time [66,67]. As such, it is clear that further research on the precise role of neutrophil apoptosis during pneumovirus infections is highly needed.

## 5. Macrophage Apoptosis

Macrophages are observed in high numbers in the airways and lungs of humans and animals with pneumovirus disease [4,18,19,20,22,23,52,54]. Macrophages are key innate immune cells acting in pathogen surveillance and initiation and resolution of inflammation through mechanisms involving antigen processing/presentation, phagocytosis and cytokine production. Macrophage depletion in wild-type mice infected by hRSV or PVM results in increased lung viral titers and greatly affects the local cytokine production [23,68,69]. However, paradoxically in PVM-infected mice, this results in prolonged survival, whereas in hRSV challenged mice, this is associated with enhanced airway occlusion on histopathological examination [23,69], suggesting that the role of macrophages may depend on specific host-pneumovirus interactions. Nevertheless, these findings underline the importance of macrophage biology in pneumovirus infection. As such, dysregulation of (apoptotic) cell death pathways in resident and migrated macrophages in the lungs may have profound consequences for the outcome of pneumovirus infection. 

Unfortunately, very few studies have focused on macrophage apoptosis in pneumovirus disease. In the histopathological studies from Welliver *et al*., fatal hRSV disease in children was associated with relatively low numbers of caspase-3 immunoreactive inflammatory cells in the lungs, as compared to children with fatal influenza virus disease [24]. In mice challenged with PVM, we observed cleaved caspase-3 positive macrophages in the alveolar spaces (unpublished observations, [70]); however, the exact magnitude and role of apoptosis of these cells in this model is not yet clear. 

*Ex vivo* and autopsy studies have shown that virus antigen is detected in macrophages in the airways and lungs of hRSV infected patients [18,71]. In addition, isolated peripheral and cord blood monocytes, as well as alveolar macrophages, are susceptible to hRSV infection *in vitro* [72,73], and PVM was recently shown to replicate in primary mouse macrophage culture [69]. Interestingly, hRSV presence in human monocytes and mouse macrophages results in decreased (susceptibility of) apoptosis, associated with decreased caspase-3 activity and enhanced expression of anti-apoptotic members of the Bcl-2 protein family and inhibitor of apoptosis protein (IAP) family [74,75]. These observations suggest pneumoviruses exploit strategies to escape apoptosis in macrophages and could explain the apparent paucity of evidence for macrophage apoptosis in the aforementioned histopathology studies. 

## 6. Apoptosis-Based Pharmacological Intervention

During the last decades, much progress has been made in the development of apoptosis-based therapeutic agents, including antisense oligodeoxynucleotides, small interfering (si)RNA, peptides/proteins and antibodies (reviewed by Fischer *et al.* [76]). Much attention in this field has been directed towards cancer treatment, however inflammatory and infectious diseases are of emerging focus. The increasing knowledge in the regulation and molecular machinery of apoptotic cell death pathways involving many different target genes and proteins has set the stage for highly promising research in pharmacological intervention. Death receptors, caspases and IAP and Bcl-2 protein family members all belong to prominent targets in current drug development [76]. However, despite the ongoing success of pre-clinical and even clinical studies exploiting the use of apoptosis-based drugs in a wide variety of diseases, many obstacles still have to be overcome, including cell-specificity and permeability, timing of intervention and potential toxicity or interference with other biological processes, such as inflammation. 

To our knowledge, up to date, no published studies have directly examined the effects of apoptosis-based pharmacological treatments in pneumovirus disease *in vivo*. Although originally designed to study macrophage depletion rather than directly focusing on apoptosis intervention, Rigaux *et al*. [69] found that intratracheal treatment with clodronate-liposomes, which induces apoptosis upon specific uptake by macrophages [77], prolongs the survival of PVM-infected mice. This suggests that enhancing macrophage apoptosis in pneumovirus disease is of clinical interest. Currently, our own group is studying the use of the irreversible pan-caspase inhibitor, z-VAD-fmk, administrated by a systemic route in mice challenged with PVM (study in progress). In addition, we have shown that treatment with DR5-Fc fusion protein, which inhibits TRAIL death receptor signaling, partly attenuates cell death of primary pediatric bronchial airway cells exposed to bronchoalveolar lavage fluid from hRSV-infected children *in vitro* [42]. However the effects of this compound *in vivo* are yet unclear. Given the strong evidence of activation of pro- and anti-apoptotic pathways during pneumovirus infection as described in the above paragraphs, more attention towards this field is highly needed.

Several critical points need to be considered in potential apoptosis-based interventions in pneumovirus disease. First is the cell-specificity. From the present overview, it becomes clear that different cell types in the lungs may show differential apoptotic responses during pneumovirus disease. Interestingly, even within a lung cell population (e.g., epithelial cells), differential responses to a single apoptotic mediator may exist [78,79], including in the setting of pneumovirus infection [34], which further increases the complexity of intervention. This stresses the need for insight in cell-specific pro- and anti-apoptotic targets and for development of small molecule compounds and vehicles for specific local intervention. On the other hand, even without specific target cell delivery systems, animal studies modeling ARDS have shown promising beneficial effects on survival and histopathological changes by using a number of systemic or intratracheal apoptosis-based treatments, including blockade of FasL by decoy receptor-3 [80], Fas:Ig fusion protein [81] or Fas-siRNA [82] and pan-caspase inhibition by z-VAD-fmk [83]. These studies successfully aimed to inhibit lung epithelial cell apoptosis during the process of lung injury. However, it remains to be elucidated whether such strategies also effectively reduce lung epithelial cell death in pneumovirus disease and, subsequently, to what extent this affects viral replication and, most importantly, clinical outcome. Second, given the dynamic course of anti- and pro-apoptotic signaling in lung epithelial cells, timing of apoptotic interventions may be critical, as, for example, inhibitory strategies may be beneficial in the late severe phase of disease, but detrimental in the early stages of viral replication. Third, concurrent iatrogenic treatments with pro-apoptotic effects, such as mechanical ventilation and/or oxygen therapy [70,84], may interact with apoptosis-based interventions in the lungs. Fourth and finally, the effect of modulating apoptosis in the lungs needs to be considered with special focus on age, as apoptosis is an important event in lung development and maturation [85].

## 7. Conclusion

Pneumovirus infection is associated with both pro- and anti-apoptotic signaling in the lungs, depending on the cell-type, involvement of specific cell death pathway and timing during the course of infection. The balance between these events is likely to be critical for the development and outcome of pneumovirus disease. However, more research is needed to fully understand the dynamic character of apoptosis during this important respiratory illness. In particular, we need to address which factors favor the viral propagation *versus* host defense. Here, we have reviewed the existing literature on this topic and have speculated on future apoptosis-based pharmacological treatments.
